# Uncovering the connection between tunicamycin-induced respiratory deficiency and reduced fluconazole tolerance in *Candida glabrata*

**DOI:** 10.3389/fmicb.2025.1528341

**Published:** 2025-04-28

**Authors:** Lijun Zheng, Yubo Dong, Jing Wang, Maoji Zhang, Yi Xu, Linfeng Ma, Liangsheng Guo

**Affiliations:** ^1^Department of Ultrasound Medicine, The Second Affiliated Hospital of Soochow University, Suzhou, China; ^2^Department of Pharmacy, The 960th Hospital of PLA, Jinan, China; ^3^Department of Pharmacy, Zibo Zhoucun People’s Hospital, Zibo, China; ^4^Jinzhou Medical University Graduate Training Base (The 960th Hospital of PLA), Jinan, China; ^5^Department of Obstetrics and Gynecology, The Second Affiliated Hospital of Soochow University, Suzhou, China

**Keywords:** *Candida glabrata*, fluconazole tolerance, fluconazole resistance, petite, tunicamycin

## Abstract

**Introduction:**

*Candida glabrata* is a prevalent opportunistic fungal pathogen in humans, and fluconazole (FLC) is one of the most commonly used antifungal agents. However, the molecular mechanisms underlying FLC tolerance in *C. glabrata* remain largely unexplored.

**Objective:**

This study aims to identify novel mechanisms regulating FLC tolerance, with a particular focus on tunicamycin (TUN)-induced respiratory deficiency.

**Methods:**

We employed three distinct experimental approaches to investigate the impact of TUN on FLC tolerance: (1) co-treatment with TUN and FLC, (2) exclusive exposure to TUN, and (3) induction of petite formation through alternative methods. Additionally, gene expression analyses were conducted to evaluate the regulation of key genes involved in the ergosterol biosynthesis pathway.

**Results:**

Our findings reveal that TUN exposure significantly abolishes FLC tolerance in *C. glabrata*, primarily through the induction of petite formation, which is characterized by mitochondrial dysfunction. Notably, TUN treatment resulted in the downregulation of critical ergosterol biosynthesis genes, including *ERG1* and *ERG11*, indicating a metabolic shift in response to endoplasmic reticulum (ER) stress. Furthermore, both TUN-induced and ethidium bromide-induced petites displayed cross-resistance to TUN and FLC but showed reduced tolerance to FLC.

**Conclusion:**

These results underscore the pivotal role of TUN-induced ER stress in modulating FLC tolerance via respiratory deficiency and alterations in ergosterol metabolism. Our study emphasizes the importance of mitochondrial integrity in maintaining drug tolerance in *C. glabrata* and suggests potential therapeutic strategies targeting metabolic pathways associated with antifungal tolerance. A deeper understanding of these mechanisms may enhance our capacity to effectively combat fungal infections.

## Introduction

The incidence of opportunistic fungal infections has been steadily increasing in recent years, particularly among immunocompromised individuals such as those with HIV/AIDS, cancer patients undergoing chemotherapy, and organ transplant recipients. Among these pathogens, *Candida* species are significant contributors to morbidity and mortality (Fisher et al., 2022). Epidemiological studies indicate that *C. glabrata* is one of the most frequently isolated *Candida* species in clinical settings, surpassing *C. albicans* in certain patient populations (Lamoth et al., 2018). The rise of *C. glabrata* infections is concerning, as this yeast demonstrates intrinsic resistance to many antifungal agents and exhibits an alarming capability for acquiring resistance during treatment. This shift highlights the urgent need for effective therapeutic strategies and a deeper understanding of the molecular mechanisms underlying drug tolerance and resistance (Brunke and Hube, 2013).

Fluconazole (FLC) has long been a cornerstone of antifungal therapy due to its broad-spectrum activity against various *Candida* species, including *C. glabrata*. It is widely used for both prophylactic and therapeutic purposes in treating candidiasis. However, the emergence of FLC-resistant strains poses a significant challenge to successful treatment outcomes (Lee et al., 2023).

In addition to drug resistance, characterized by elevated minimum inhibitory concentrations (MIC) of antifungal agents, a new term—antifungal tolerance—has recently been introduced to describe the ability of drug-susceptible fungal strains to grow slowly in the presence of supra-MIC concentrations of these agents (Rosenberg et al., 2018; Berman and Krysan, 2020). FLC tolerance can be assessed using disk diffusion assays, where FLC-susceptible strains exhibit an obvious zone of inhibition (ZOI). In these assays, photographs of the plates are analyzed using the *diskImageR* pipeline. The level of drug resistance is measured by the radius of ZOI (RAD), while tolerance is evaluated based on the fraction of growth (FoG) within the ZOI (Gerstein et al., 2016; Berman and Krysan, 2020). While FLC tolerance has been best studied in *C. albicans*, it is noteworthy that *C. glabrata* is more closely related to *Saccharomyces cerevisiae* than to other *Candida* species, sharing significant genetic and evolutionary similarities. This relationship sets *C. glabrata* apart within the *Candida* genus and influences our understanding of its biology, pathogenicity, and response to antifungal treatments (Roetzer et al., 2011).

We have recently demonstrated that FLC tolerance exists in wild-type *C. glabrata* isolates and can be induced by exposure to FLC. Furthermore, similar to findings in *C. albicans*, we showed that FLC tolerance in *C. glabrata* is dependent on the heat shock protein Hsp90 and calcineurin (Zheng et al., 2024b). However, the factors modulating FLC tolerance in *C. glabrata* remain largely unknown.

Tunicamycin (TUN) is a widely used inducer of endoplasmic reticulum (ER) stress. TUN inhibits UDP-N-acetylglucosamine-dolichol phosphate N-acetylglucosamine-1-phosphate transferase (GPT), thereby blocking the initial step of glycoprotein biosynthesis in the ER. This inhibition leads to the accumulation of unfolded glycoproteins in the ER, triggering ER stress (Lee, 1992). TUN has been linked to drug resistance in both mammalian and yeast cells. Specifically, the inhibition of glycosylation by TUN sensitizes multidrug-resistant (MDR) gastric cancer cells to TUN-induced cell death (Wu et al., 2018), as well as enhancing the sensitivity of MDR cell lines, such as NIH-3T3 and KB-8-5-11, to a range of chemotherapeutic agents (Hiss et al., 1996). In the diploid fungal pathogen *Candida albicans*, TUN induces amplification of chromosome 2, which results in the upregulation of several genes associated with tolerance to caspofungin, the first-line antifungal drug. This genetic adaptation potentiates cross-tolerance to both TUN and caspofungin (Yang et al., 2021). In the haploid fungal pathogen *Cryptococcus neoformans*, TUN induces formation of multiple aneuploid karyotypes, and some aneuploids, including disomy of chromosome 1 and chromosome 4, are cross-resistant to TUN and FLC (Zheng et al., 2024a). Previous studies have demonstrated that TUN can serve as an adjuvant to eliminate FLC tolerance in *C. albicans*; however, the underlying mechanism remains unexplored (Rosenberg et al., 2018). In this study, we investigated the effect of TUN on FLC tolerance in *C. glabrata* and sought to elucidate the mechanisms involved.

## Materials and methods

### Strains and growth conditions

The *C. glabrata* FLC-tolerant isolates CG4 and CG8, and the non-tolerant reference strain BG2 served as the progenitors for this study. The profile of FLC tolerance in CG4 has been detailed in our previous report (Zheng et al., 2024b). Stock cultures were preserved in 25% glycerol and stored at −80°C. Cells were routinely cultured in Yeast Extract-Peptone-Dextrose (YPD) medium, which contains 1% (w/v) yeast extract, 2% (w/v) peptone, and 2% (w/v) D-glucose, at 30°C using a shaking incubator set to 150–200 rpm. For YPG medium, the composition included 1% (w/v) yeast extract, 0.2% (w/v) peptone, and 3% (w/v) glycerol, with 2% (w/v) agar added for solid media. Drug solutions were prepared in dimethyl sulfoxide (DMSO) and stored at −20°C.

### Disk diffusion assay

Disk diffusion assays were performed according to the protocols outlined in our previous studies (Guo et al., 2024; Zheng et al., 2024b; Zheng et al., 2024c), following the CLSI M44-A2 guidelines for antifungal disk diffusion susceptibility testing (CLSI, 2009), with minor modifications. Briefly, strains were streaked from glycerol stocks onto YPD agar plates and incubated at 30°C for 48 h. Colonies were then suspended in distilled water and adjusted to a concentration of 1 × 10^6^ cells/mL. A volume of 100 μL of this cell suspension was evenly spread across YPD plates. An empty paper disk (6 mm diameter and 0.7 mm thickness) was saturated with 5 μL of 40 mg/mL FLC and placed at the center of each plate. The plates were subsequently incubated at 30°C and photographed after 48 h. The analysis of the disk diffusion assay was conducted using the *diskImageR* pipeline (Gerstein et al., 2016), measuring parameters such as the fraction of growth within the zone of inhibition (FoG) and the radius of inhibition (RAD).

### Selection of colonies from the inhibition zone on YPD + TUN plates

For the isolation process, cells were suspended in distilled water and carefully adjusted to a concentration of 1 × 10^6^ cells/mL. Subsequently, 100 μL of this cell suspension was evenly spread onto a YPD plate supplemented with 1 μg/mL TUN. An empty paper disk saturated with 5 μL of 40 mg/mL FLC was placed at the center of the plate.

Following an incubation period of 48 h at 30°C, four colonies were randomly selected from within the ZOI for further examination. These chosen colonies were streaked onto fresh YPD plates and underwent an additional 48-h incubation. From each replicate, a single colony was then meticulously chosen to progress to the subsequent stage of meticulous analysis and exploration.

### Acquiring adaptors through elevated tunicamycin concentrations

The cells were suspended in distilled water and adjusted to a concentration of 1 × 10^7^ cells/mL. Subsequently, 100 μL of this cell suspension was evenly spread on YPD plates supplemented with TUN. The plates were then incubated at 30°C for a duration of 5 days, after which adaptors were randomly chosen from the drug-treated plates.

### Spot assay

Cells were suspended in distilled water and adjusted to a concentration of 1 × 10^7^ cells/mL. A volume of 3 μL of the cell suspension was spotted onto YPD or YPG plates. For testing susceptibility to TUN, 3 μL of 10-fold serial dilutions were spotted on YPD plates containing 8 μg/mL TUN. The plates were incubated at 30°C and photographed after 48 h.

### Induction of petite formation using ethidium bromide

The technique for inducing petite formation with Ethidium bromide (EtBr) was adapted from Fox et al. (1991) with slight modifications. Thawed test strains were streaked onto YPD plates and incubated at 30°C for 48 h. A single colony was then inoculated into YPD broth with 25 μg/mL EtBr, followed by transfer to a second culture with the same medium. Saturated cultures were streaked onto YPD plates to isolate colonies, which were subsequently streaked onto YPD and YPG plates to confirm respiratory deficiency.

### RNA extraction, synthesis of complementary DNA and quantitative real-time PCR

To compare between progenitor and petite strains, they were cultured in YPD broth until reaching the logarithmic phase (OD_600_ = 1.0). To assess the effect of TUN on gene expression, the logarithmic phase cultures were split into two groups. One group received 8 μg/mL TUN supplementation, while the other was supplemented with an equivalent amount of vehicle. After a 3-h incubation period, the cells were harvested by centrifugation.

Total RNA was extracted using YeaStar RNA kit (Zymo Research) following the manufacturer’s guidelines. The RNA concentration and purity were evaluated with a spectrophotometer (NanoDrop 2000C; ThermoFisher Scientific) through absorbance measurements at 230 nm (OD_230_), 260 nm (OD_260_), and 280 nm (OD_280_). Additionally, RNA integrity was confirmed by electrophoresis on 1% denaturing and non-denaturing agarose gels in selected samples.

The RNA samples were treated with DNase I (ThermoFisher Scientific) at 37°C for 30 min following the manufacturer’s protocol. Approximately 1 μg of total RNA underwent reverse transcription (RT) using High Capacity cDNA Reverse Transcription Kit (ThermoFisher Scientific).

The expression of candidate genes was quantified by real-time RT-PCR using the CFX96 Touch Real-Time PCR system (Bio-Rad). The housekeeping *ACT1* was used as internal control. The relative quantification of gene expression was performed by the 2^−ΔΔCT^ method (Schmittgen and Livak, 2008). Each reaction was performed in triplicate, and mean values of relative expression were determined for each gene. Primers are listed in Supplementary Table S1.

### Measurement of FLC minimal inhibitory concentration

The experiment was performed according to the Clinical and Laboratory Standards Institute (CLSI) recommendations (CLSI, 2017) with slight modifications. Briefly, yeast cells were harvested during the logarithmic growth phase, washed twice with sterile distilled water, and resuspended in distilled water. The cell density was adjusted to a final concentration of 2.5 × 10^3^ cells/mL in YPD broth supplemented with fluconazole (FLC) at concentrations ranging from 0.125 to 128 μg/mL. The cell suspensions were then aliquoted into 96-well microtiter plates, with each well containing 200 μL of the suspension. The plates were incubated at 30°C for 24 h under static conditions. After incubation, the optical density at 600 nm (OD600) was measured using a microplate reader to quantify cell growth. Each condition was tested in triplicate to ensure reproducibility, and control wells containing YPD broth without FLC were included to account for background growth.

### Multilocus sequence typing

Multilocus sequence typing (MLST) analysis was conducted as previously described by Dodgson et al. (2003). Six loci (*FKS*, *LEU2*, *NMT1*, *TRP1*, *UGP1*, and *URA3*) were amplified using the primers specified in Dodgson et al. (2003). PCR reactions were carried out in 20-μL volumes containing 5 ng of genomic DNA, 10 μL of 2 × Phusion Green Hot Start II High-Fidelity PCR Master Mix (Fisher Scientific), and 0.1 μM of each primer. The amplified products were sequenced bidirectionally (forward and reverse) using the same primers as those employed for the PCR amplification.

### Statistical analysis

All disk diffusion assays represent the average of three technical replicates, with error bars indicating the standard deviation. Statistical analyses were conducted using a two-tailed Student’s *t*-test in Microsoft Excel. A *p*-value of less than 0.05 was considered statistically significant. ** indicates *p* < 0.01, and *** indicates *p* < 0.001.

## Results

### Tunicamycin disrupts fluconazole tolerance in clinical isolates of *Candida glabrata* without affecting resistance

In our study, we evaluated the effects of TUN on the FLC tolerance of three clinical isolates of *C. glabrata*. Each isolate exhibited a notable tolerance to FLC, as evidenced by the presence of significant lawn growth within ZOI. In a pilot experiment, we tested the effect of various concentrations of TUN on FLC tolerance in CG4. We found that 0.5 μg/mL of TUN did not abolish FLC tolerance, whereas 1 and 2 μg/mL concentrations effectively eliminated FLC tolerance. At 4 μg/mL, the growth of most cells on the plate was inhibited (Supplementary Figure S1). Based on these results, we selected 1 μg/mL of TUN to assess its impact on FLC tolerance in CG4, as well as in two other *C. glabrata* isolates, CG8 and CG10.

**Figure 1:**
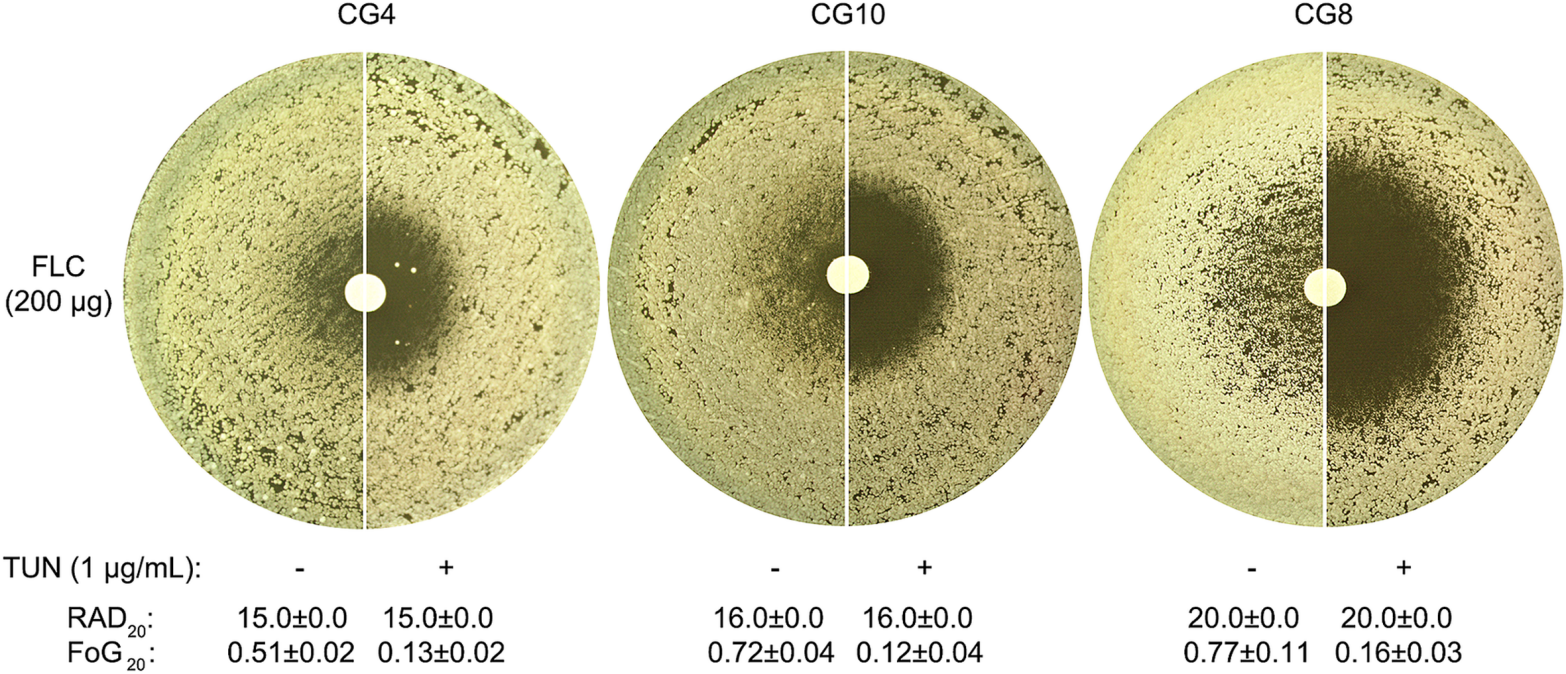
Impact of tunicamycin on fluconazole tolerance and resistance in *C. glabrata*. Top Panel: Three clinical isolates of *C. glabrata* were tested on YPD agar plates, both with and without the addition of TUN. Each disk contained 200 μg of FLC. The plates were incubated at 30°C for 48 h before being photographed to assess the growth response. Bottom Panel: Photographs were edited using ImageJ prior to quantification with the R package *diskImageR*. The images were cropped to a uniform size, colors were inverted, and brightness and contrast were adjusted using consistent parameters across all images to enhance the contrast between the white disk and black background. Susceptibility was measured as RAD_20_, the radius where 20% reduction of growth occurs, while tolerance was measured as FoG_20_, the fraction of growth above RAD_20_. The RAD_20_ and FoG_20_ values shown represent the means ± standard deviation of three biological replicates for each isolate.

Upon the supplementation of 1 μg/mL TUN, a marked change in the response was observed in all the 3 isolates: the ZOI became clear, indicating a loss of FLC tolerance (Figure 1, top panel).

Quantitative assessments revealed that a concentration of TUN at 1 μg/mL led to a significant decrease of FoG_20_ values across all three tested isolates, with statistical significance confirmed (*p* < 0.001, two-tailed Student’s *t*-test). Interestingly, while TUN effectively diminished FLC tolerance, it did not appear to affect the RAD_20_ (Figure 1, bottom panel), indicating that TUN’s mechanism of action primarily targets pathways associated with tolerance rather than directly impacting resistance mechanisms.

### Emergence of respiratory-deficient mutants in *Candida glabrata* driven by combined stress from tunicamycin and fluconazole treatment

**Figure 2:**
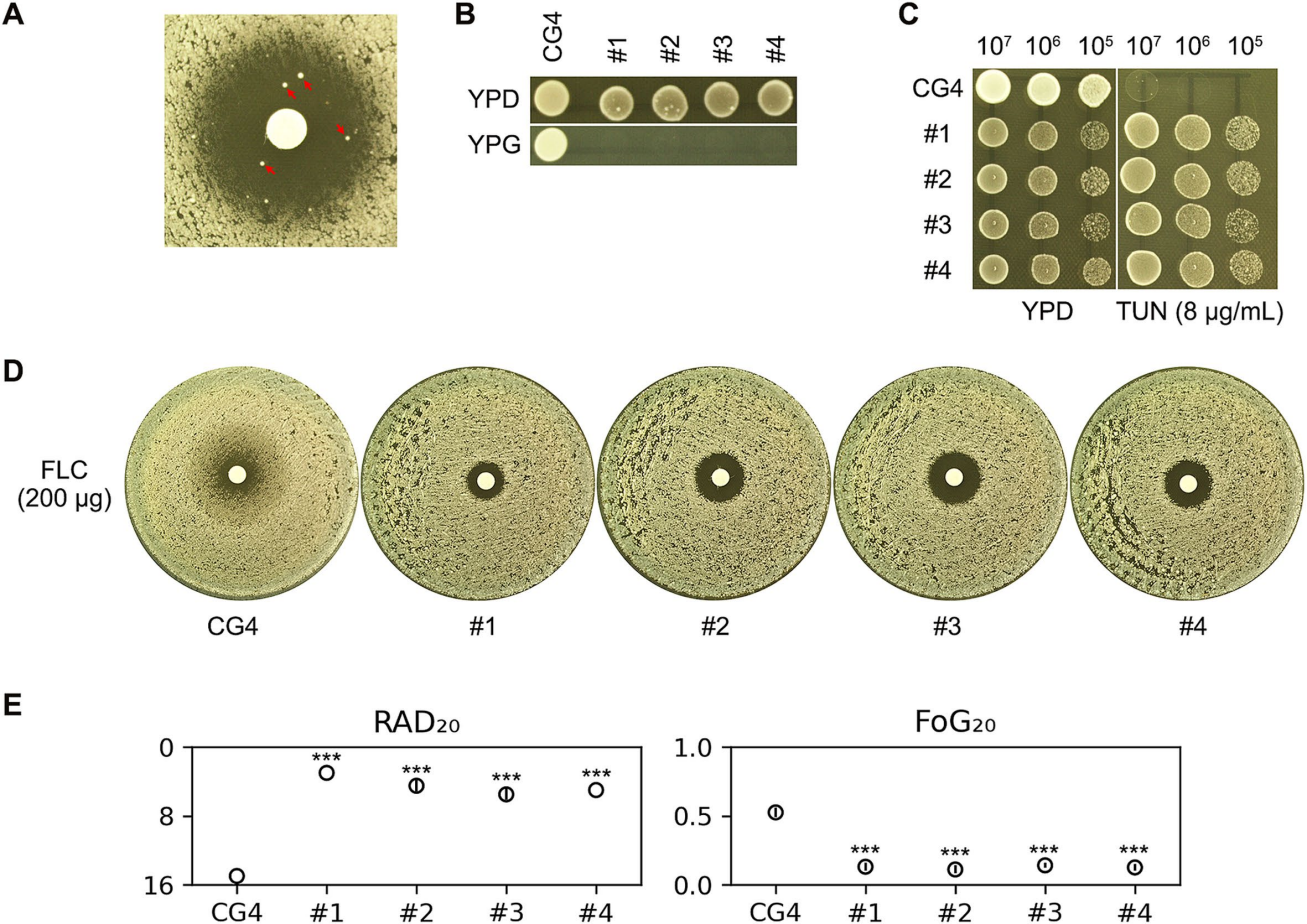
Assessment of antifungal susceptibility and growth characteristics in CG4 and derived colonies under tunicamycin and fluconazole treatment. **(A)** The CG4 isolate was subjected to a disk diffusion assay using disks containing 200 μg of FLC. The assay was conducted on YPD medium supplemented with 1 μg/mL of TUN. After a 48-h incubation period, we observed the emergence of several unusually large colonies within ZOI, which were clearly distinguishable. Four of these colonies, indicated by red arrows in the image, were selected for further analysis. **(B)** To investigate the growth characteristics of the progenitor strain CG4 and the selected four colonies (#1-#4), we performed spot assays on both YPD and YPG plates. The YPD plates utilize glucose as the carbon source, while the YPG plates utilize glycerol. This comparison allowed us to assess the respiratory capabilities of these colonies in different nutrient contexts. **(C)** A spot assay was executed on both YPD and YPD plates supplemented with 8 μg/mL TUN. For this assay, 3 μL of 10-fold serial dilutions of each strain were spotted onto the plates. This method enabled us to evaluate the growth patterns and stress responses of each isolate in the presence of TUN. **(D)** In parallel, another disk diffusion assay was performed utilizing disks impregnated with 200 μg of FLC. This experiment aimed to quantify the antifungal susceptibility of the isolates, providing further insight into their resistance profiles. **(E)** The images obtained from the disk diffusion assay plates were quantified using the *diskImageR* package. Results are presented as the mean and standard deviation from three biological replicates for each isolate. Statistical analysis was conducted using a two-tailed Student’s *t*-test to determine significance. Asterisks denote statistical significance, with *** indicating *p* < 0.001 when compared to the progenitor strain CG4. For all assays, the plates were incubated at 30°C for 48 h prior to photography.

While TUN effectively abolished FLC tolerance in our experiments, we noted an intriguing phenomenon during the testing of the CG4 isolate; a few exceptionally large colonies were observed within ZOI, as indicated by red arrows in Figure 2A. To further investigate this anomaly, we randomly selected four of these colonies, designated as #1 through #4, for analysis.

Interestingly, although the progenitor strain CG4 was capable of growing on YPG plate—where glycerol served as the carbon source—none of the four chosen colonies exhibited growth under the same conditions. This observation suggests that these colonies may be petites, which are characterized by respiratory deficiencies due to defects in mitochondrial function (Figure 2B).

To assess the impact of TUN on these four colonies, we conducted a spot assay, which revealed that each of the four colonies demonstrated improved growth compared to the parental CG4 strain when exposed to TUN (Figure 2C). This finding indicates a possible adaptive response or compensatory mechanism in the petites that enhances their proliferation in the presence of TUN.

Furthermore, results from a disk diffusion assay using disks containing FLC showed that all four colonies exhibited clear ZOI with reduced overall size (Figure 2D). Quantitative analysis of the disk diffusion assay images confirmed that all four colonies had significantly lower values for both FoG_20_ and RAD_20_, with statistical significance denoted (*p* < 0.001, two-tailed Student’s *t*-test). These results collectively indicate that the four colonies not only lost FLC tolerance but also gained FLC resistance (Figure 2E).

### Tunicamycin-induced petite formation alters fluconazole tolerance and resistance

**Figure 3:**
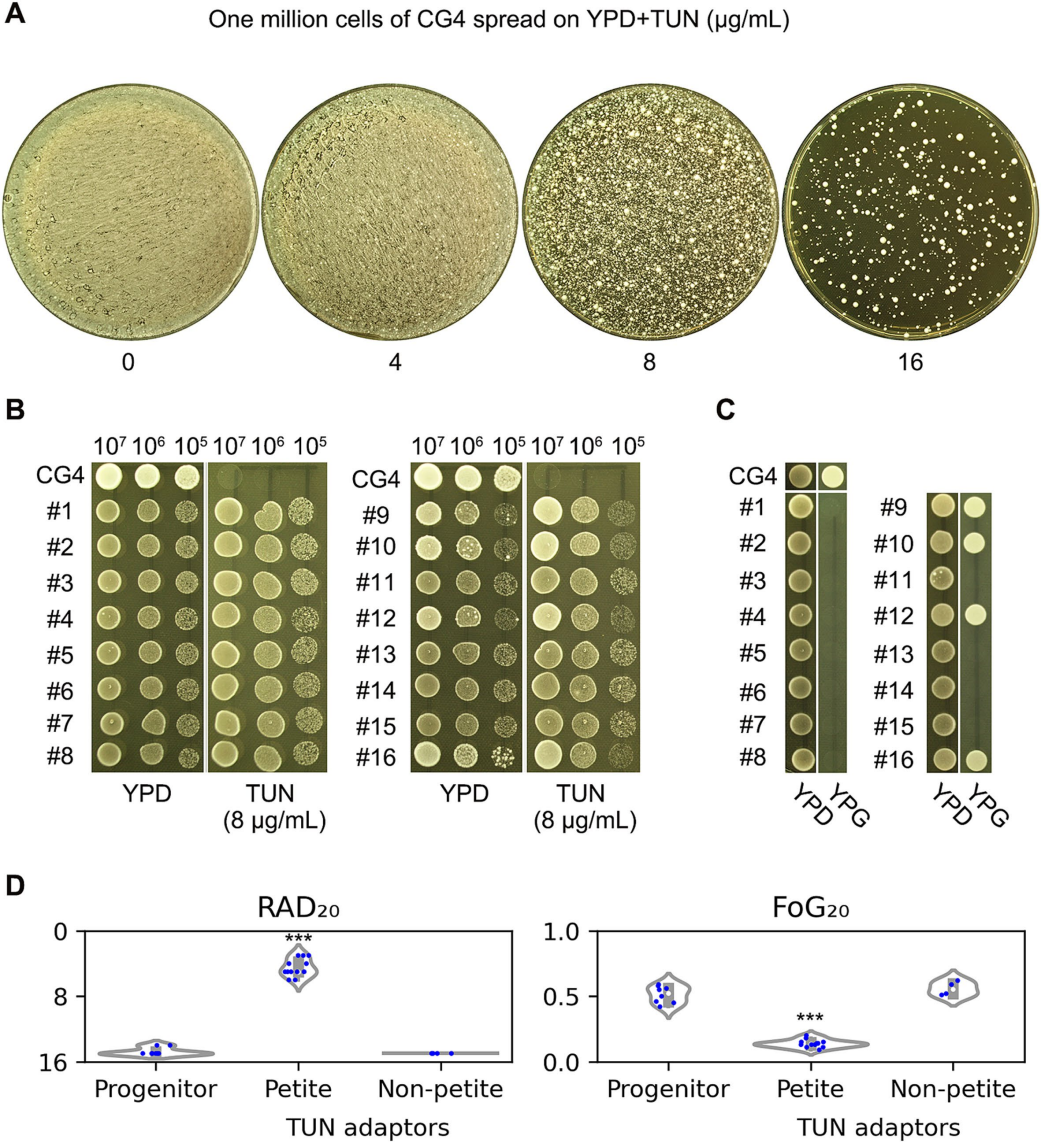
Impact of tunicamycin on fluconazole susceptibility and respiratory proficiency. **(A)** Cells of the CG4 strain were suspended in distilled water and adjusted to a concentration of 1 × 10^7^ cells/mL. A volume of 100 μL of this cell suspension was then spread onto YPD plates supplemented with 4–16 μg/mL TUN. The plates were incubated at 30°C for 3 days before being photographed. From the plate containing 16 μg/mL TUN, 16 randomly selected colonies (referred to as “adaptors”) were chosen for further analysis. **(B)** Both the progenitor strain and the TUN adaptors were evaluated for resistance to TUN using a spot assay. For each strain, cells were adjusted to 5 × 10^7^ cells/mL. A volume of 3 μL from 10-fold serial dilutions of each strain was spotted onto YPD plates with or without 8 μg/mL TUN. The plates were incubated at 30°C for 48 h and subsequently photographed. **(C)** The adaptors were assessed for respiratory proficiency. For each strain, cells were again adjusted to 5 × 10^7^ cells/mL using distilled water, and 3 μL were spotted onto both YPD and YPG plates. After incubation at 30°C for 48 h, the plates were photographed. Among the adaptors, four—specifically #9, #10, #12, and #16—were able to grow on YPG, while the remaining 12 adaptors were unable to do so. **(D)** To evaluate susceptibility to FLC, a disk diffusion assay was performed using disks containing 200 μg of FLC. The resulting images were analyzed using the *diskImageR* package. Results are presented as the mean of three biological replicates for each strain. Statistical analysis was conducted using a two-tailed Student’s *t*-test to assess significance. Asterisks denote statistical significance, with *** indicating *p* < 0.001 when compared to the progenitor strain CG4.

In the experiments described above, the progenitor strain CG4 was exposed to a combination of TUN and FLC. In this section, we investigate the impact of TUN alone on the CG4 isolate. To assess this, CG4 cells were spread on YPD plates containing varying concentrations of TUN. Notably, on the plate with 16 μg/mL TUN, several hundred colonies emerged, which we refer to as “adaptors.” In contrast, lower concentrations of TUN resulted in a uniform lawn growth across the plates (Figure 3A).

From the colonies that appeared at the highest concentration, we randomly selected 16 adaptors for further analysis. These adaptors were then compared to the progenitor strain in terms of their resistance to TUN. A spot assay demonstrated that all 16 adaptors were capable of growing in the presence of 8 μg/mL TUN, while the progenitor strain exhibited marked inhibition of growth under the same conditions (Figure 3B). Thus, all the 16 adaptors gained resistance to TUN.

Next, we evaluated the ability of these adaptors to utilize glycerol as a carbon source, which is indicative of respiratory competency. Of the 16 adaptors tested, four—specifically #9, #10, #12, and #16—were able to grow on YPG plates, suggesting that the majority of the adaptors (12 out of 16) exhibited respiratory deficiencies (Figure 3C).

Lastly, we assessed the susceptibility of the adaptors to FLC. The petite adaptors demonstrated significantly RAD_20_ and FoG_20_ compared to the progenitor strain, indicating that they had lost FLC tolerance and gained FLC resistance. In contrast, the non-petite adaptors did not show significant changes in RAD_20_ or FoG_20_ when compared to the progenitor (Figure 3D).

Susceptibility to FLC was further assessed by determining the minimum inhibitory concentration (MIC). The progenitor strain and non-petite TUN adaptors exhibited MICs of 16 μg/mL, whereas the petite TUN adaptors showed significantly higher MICs, ranging from 64 to 128 μg/mL.

Besides CG4, another isolate, CG8, was also tested. We found that TUN at a concentration of 8 μg/mL significantly inhibited the growth of CG8 (Supplementary Figure S2A). Twelve adaptors (#1-#12) were randomly selected, and three of them (#3, #8, and #9) failed to grow on YPG plates (Supplementary Figure S2B). Disk diffusion assays showed that the petite adaptors had significantly lower FoG_20_ and smaller RAD_20_ values compared to the wild-type (*p* < 0.001, two-tailed Student’s *t*-test), while the non-petite adaptors did not exhibit significant changes in FoG_20_ or RAD_20_ (*p* > 0.05, two-tailed Student’s *t*-test).

In addition to the two clinical isolates, the reference strain BG2 was also tested. We found that TUN at 8 μg/mL significantly inhibited BG2 (Supplementary Figure S3A). From a pool of randomly selected adaptors (#1-#30), eight were identified as petites (Supplementary Figure S3B). Both the progenitor BG2 strain and the non-petite TUN adaptors exhibited clear zones of inhibition (ZOI) with similar values for RAD and FoG. In contrast, the petite adaptors showed no detectable ZOI, indicating a high level of resistance to FLC (Supplementary Figures S4C,D).

### Characterization of EtBr-evolved petites: similar phenotypes of altered fluconazole susceptibility

From the experiments described above, we established a connection between respiratory deficiency and resistance to TUN, as well as altered susceptibility to FLC, characterized by decreased tolerance and increased resistance. Notably, these petites were selected either through exposure to TUN alone or a combination of TUN and FLC. To explore whether petites selected under different stress conditions exhibit similar phenotypes, we turned our attention to Ethidium Bromide (EtBr). EtBr is known to inhibit mitochondrial DNA (mtDNA) synthesis and induce degradation of pre-existing mtDNA, leading to the conversion of respiratory-sufficient yeast into respiratory-deficient petites (Goldring et al., 1970).

**Figure 4:**
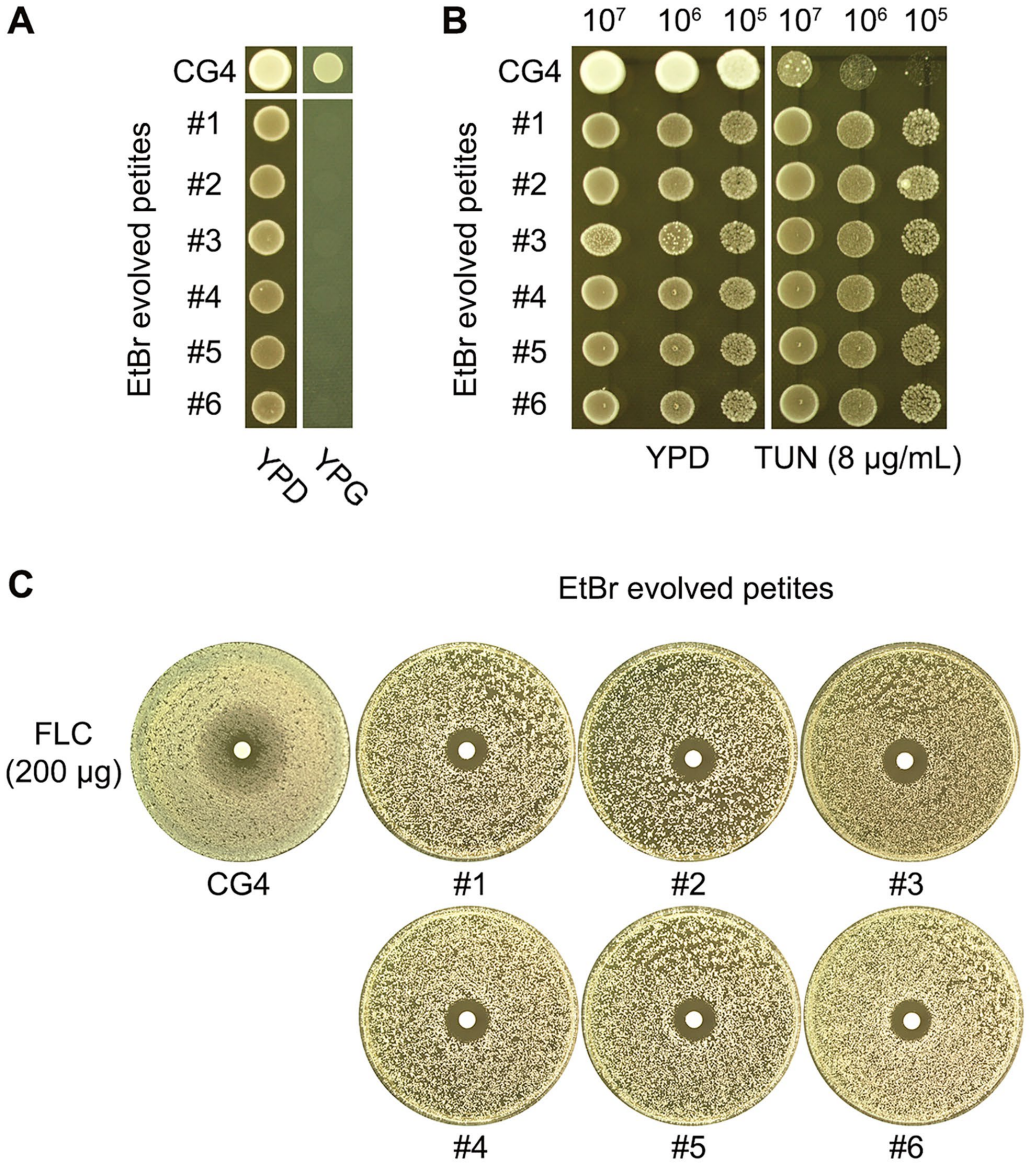
Characterization of EtBr-evolved petites: altered susceptibility to TUN and FLC. CG4 was pre-grown in YPD broth supplemented with Ethidium Bromide (EtBr) to induce the formation of respiratory-deficient petites. Six randomly selected colonies from this culture, labeled #1 through #6, were subsequently spotted onto both YPG and YPD plates to assess their respiratory proficiency **(A)**. To further investigate the phenotypic characteristics of the EtBr-evolved petites, we compared their susceptibility to TUN and FLC against the progenitor strain. Susceptibility to TUN was assessed using a spot assay **(B)**, which demonstrated that the EtBr-evolved petites (#1-#6) showed enhanced resistance to TUN compared to the progenitor CG4. Similarly, for FLC susceptibility, a disk diffusion assay was performed **(C)**. The results indicated that all six petites exhibited smaller ZOI compared to the progenitor, suggesting a loss of FLC tolerance and an increase in FLC resistance.

In our study, CG4 was cultured in YPD broth supplemented with EtBr. After 24 h of incubation, the culture was diluted and subsequently spread onto YPD plates. From this plating, six randomly selected colonies were tested for their ability to grow on YPG, which serves as an indicator of respiratory competency. None of the selected colonies could grow on YPG, confirming that all were indeed petites (Figure 4A). We designated these colonies as “EtBr-evolved petites.”

Further analysis using a spot assay revealed that the EtBr-evolved petites exhibited superior growth compared to the progenitor strain in the presence of 8 μg/mL TUN, indicating a level of resistance to TUN (Figure 4B). Additionally, results from a disk diffusion assay demonstrated that all six petites had clear but smaller ZOI in comparison to the progenitor strain, suggesting that they lost FLC tolerance while gaining FLC resistance (Figure 4C).

### Petites have increased expression of efflux genes and reduced expression of *ERG* genes

**Figure 5:**
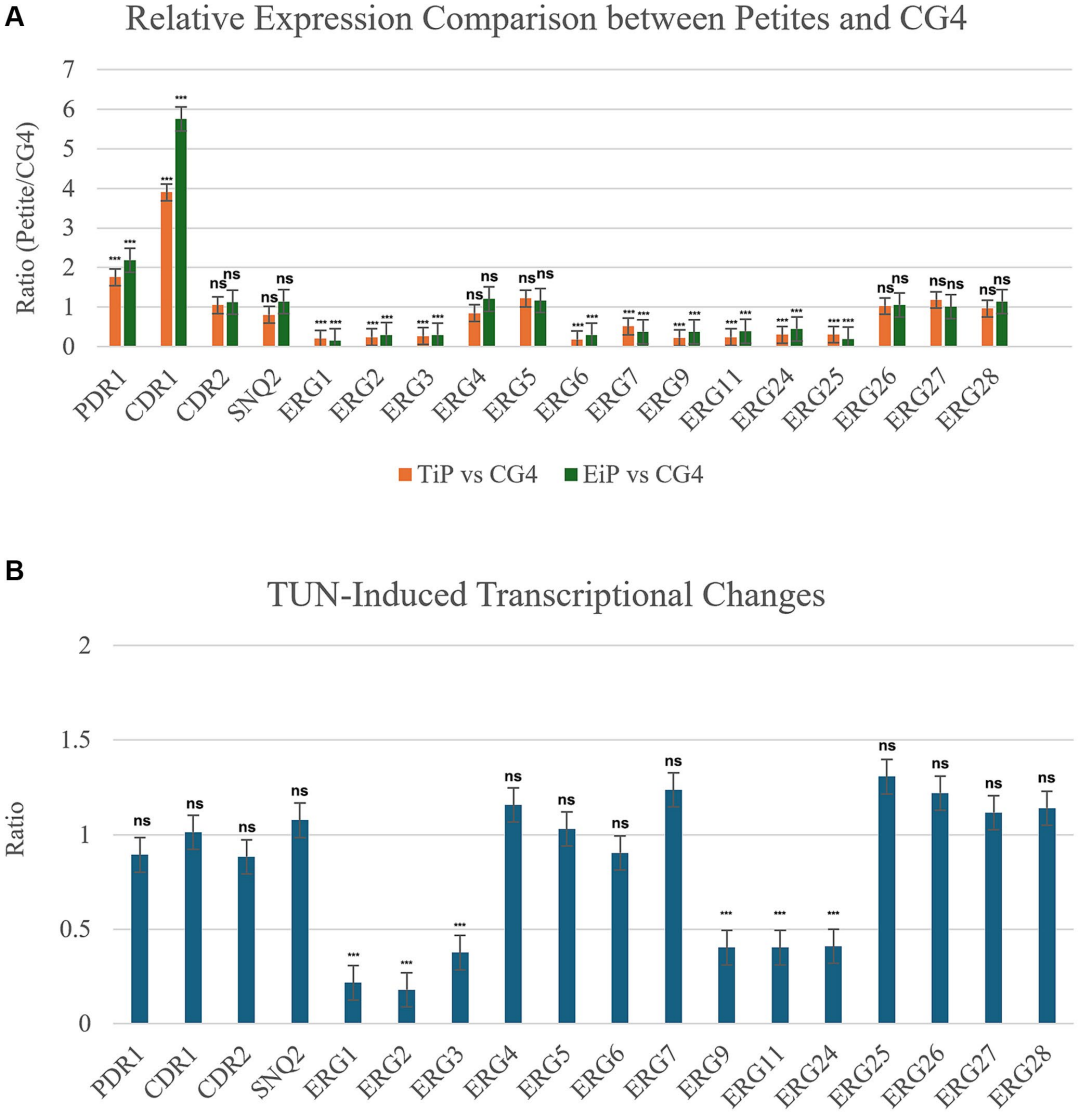
Relative expression analysis of efflux and ERG genes. **(A)** Comparison of gene expression between petite strains and progenitor CG4. Two petites were examined, TiP representing TUN-induced petite and EiP representing EtBr-induced petite. Cells were cultured to logarithmic phase, and the relative expression levels of genes in petites compared to CG4 are displayed in the figure. **(B)** Impact of TUN on gene expression. CG4 cells were grown to logarithmic phase and then treated with 8 μg/mL TUN. The graph illustrates the relative expression levels of genes in TUN-treated cells compared to vehicle-treated cells. In both A and B, the housekeeping *ACT1* was used as internal control. The relative quantification of gene expression was performed by the 2^−ΔΔCT^ method. Each reaction was performed in triplicate, and mean values of relative expression were determined for each gene.

Resistance to azoles typically arises from increased efflux and alterations in the target (Lee et al., 2023). In the *C. glabrata* genome, drug efflux is primarily mediated by ATP-binding cassette transporters, particularly through the *C. glabrata* sensitivity to 4-Nitroquinoline N-oxide (*CgSNQ2*) and *C. glabrata* Drug Resistance 1 and 2 (*CgCDR1* and *CgCDR2*) genes (Hassan et al., 2021). Moreover, *PDR1* encodes the central transcription factor that triggers the expression of *CDR1* (Moye-Rowley, 2020). Brun et al. identified that resistance in FLC-induced *C. glabrata* petites was attributed to the upregulation of efflux genes, particularly *CDR1* (Brun et al., 2004). In *Saccharomyces cerevisiae*, a model yeast closely related to *C. glabrata*, various respiratory inhibitors have diverse impacts on ergosterol biosynthesis (Adams and Parks, 1969). We hypothesize that TUN-induced and EtBr-induced petites might influence the expression of efflux and/or *ERG* genes, thereby enhancing FLC resistance while reducing FLC tolerance. Consequently, we compared the gene expressions between two petites and the parent CG4. One petite was induced by TUN exposure (TUN-induced petite, TiP), while the other was induced by EtBr exposure (EtBr-induced petite, EiP). Our findings revealed a significant increase in the expression of *CDR1* and *PDR1* compared to CG4 (*p* < 0.001, two-tailed Student’s *t*-test), whereas the expression of most ERG genes, including *ERG1*, *ERG2*, *ERG3*, *ERG6*, *ERG7*, *ERG9*, *ERG11*, *ERG24*, *ERG25*, was significantly reduced (*p* < 0.001, two-tailed Student’s *t*-test) in both types of petites (Figure 5A).

Next, we investigated the effect of TUN exposure on the expression of efflux and *ERG* genes. Exposure of CG4 to 8 μg/mL of TUN significantly down-regulated multiple *ERG* genes, including *ERG1*, *ERG2*, *ERG3*, *ERG9*, *ERG11*, and *ERG24* (*p* < 0.001, two-tailed Student’s *t*-test). However, TUN had negligible effects on the expression of *CDR1*, *CDR2*, *SNQ2*, and *PDR1* (*p* > 0.05, two-tailed Student’s *t*-test) (Figure 5B).

## Discussion

Previous studies have established that azole tolerance in *C. albicans* is influenced by various physiological factors, including temperature, medium composition, and specific proteins such as Hsp90, calcineurin, V-ATPase, as well as aneuploidy (Xu et al., 2021; Kukurudz et al., 2022; Sun et al., 2023; Todd et al., 2023; Yang et al., 2023). Recently, we discovered that FLC tolerance in *C. glabrata* also depends on the heat shock protein Hsp90 and calcineurin (Zheng et al., 2024b). In the current study, we identify a novel factor—TUN-induced respiratory deficiency—that regulates FLC tolerance in *C. glabrata*, marking the first connection of this kind.

To assess the impact of TUN on FLC tolerance, we employed three distinct experimental approaches: (1) combining TUN with FLC, (2) exposing cells exclusively to TUN, and (3) utilizing an alternative method to induce petite formation. Our findings demonstrate that TUN can abolish FLC tolerance, primarily through the downregulation of ergosterol biosynthesis pathway genes.

TUN is a well-characterized inducer of ER stress, acting by inhibiting the enzyme UDP-N-acetylglucosamine: dolichol phosphate N-acetylglucosamine-1-phosphate transferase, which plays a critical role in the synthesis of N-linked glycans. Consequently, newly synthesized glycoproteins cannot undergo proper glycosylation, leading to the accumulation of misfolded or unprocessed proteins within the ER lumen. Cells have a limited capacity to manage this accumulation, prompting the activation of the unfolded protein response (UPR)—a cellular stress response mechanism aimed at restoring normal ER function (Lee, 1992).

Ergosterol, the major sterol found in fungal membranes, is synthesized in the ER through a complex pathway involving numerous enzymes encoded by the ERG genes in yeasts. In the *C. glabrata* genome, these *ERG* genes include *ERG9/CAGL0M07095g, ERG1/CAGL0D05940g, ERG7/CAGL0J10824g, ERG11/CAGL0E04334g, ERG24/CAGL0I02970g, ERG25/CAGL0K04477g, ERG26/CAGL0G00594g, ERG27/CAGL0M11506g, ERG28/CAGL0J02684g, ERG6/CAGL0H04653g, ERG2/CAGL0L10714g, ERG3/CAGL0F01793g, ERG5/CAGL0M07656g, ERG4/CAGL0A00429g* (Eliaš et al., 2024). Notably, *ERG1* and *ERG11* represent two rate-limiting steps in the ergosterol biosynthesis pathway (Jorda and Puig, 2020).

The primary function of the UPR is to manage protein folding and restore ER homeostasis, necessitating regulatory shifts in metabolic priorities. Our study reveals that TUN-induced ER stress leads to reduced expression of ergosterol biosynthesis genes, including the key players *ERG1* and *ERG11*. This finding suggests that when the UPR is activated due to protein misfolding, the cell reallocates resources away from sterol biosynthesis to prioritize the resolution of ER stress. This supports the notion that the UPR not only oversees protein folding but also influences metabolic pathways vital for cellular integrity.

Additionally, we identified petite formation as a primary mechanism for rapid adaptation to TUN-induced ER stress in *C. glabrata*. We also evaluated petites induced by exposure to EtBr and found that both TUN-induced and EtBr-induced petites exhibited cross-resistance to TUN and FLC, albeit with a diminished tolerance to FLC. Notably, the expression of multiple *ERG* genes, including *ERG1* and *ERG11*, was lower in both types of petites compared to the wild-type strain.

Typically, petites are associated with a loss of mitochondrial function, significantly influencing cellular metabolism. The analysis of petites induced by EtBr underscores the similarity between TUN and EtBr in promoting cross-resistance to TUN and FLC, suggesting a shared adaptive response mechanism involving mitochondrial dysfunction and altered metabolic states. The observed reduction in the expression of multiple ergosterol biosynthesis genes in both types of petites reinforces the idea that perturbations in mitochondrial function adversely affect sterol metabolism. This reduction in key ERG gene expression implies that these petites may develop compensatory mechanisms to cope with drug stress, though this comes at the cost of FLC tolerance.

Reference to Siscar-Lewin et al.’s research highlights that deletion of the mitochondrial DNA polymerase *CgMIP1* triggers loss of mitochondrial function and petite formation, which also conveys cross-resistance to TUN and FLC (Siscar-Lewin et al., 2021). Our findings build upon this work, strengthening the hypothesis that mitochondrial integrity is essential for maintaining both ergosterol biosynthesis and drug tolerance in *C. glabrata* under stress conditions.

Notably, MLST analysis indicates that CG4 belongs to ST7, the most prevalent genotype in Asia (Meng et al., 2025). Our study demonstrates that TUN can disrupt FLC tolerance and induce FLC resistance in CG4. Given the prevalence of ST7 strains in Asia, these findings are likely to have broad applicability and relevance to other ST7 strains in this region, particularly in understanding how ER stress influences antifungal tolerance and resistance. However, further studies are needed to explore whether similar mechanisms operate in other ST7 strains. Such efforts will provide deeper insights into the epidemiology and treatment of *C. glabrata* infections, particularly in regions where ST7 is dominant.

## Conclusion

In summary, our study uncovers a novel mechanism by which TUN-induced ER stress modulates FLC tolerance in *C. glabrata*. We demonstrate that this stress response leads to petite formation and reduction in ergosterol biosynthesis. This novel insight into the relationship between ER stress, mitochondrial dysfunction, antifungal resistance and tolerance underscores potential avenues for developing more effective therapeutic strategies against resistant fungal strains.

## Data Availability

The original contributions presented in the study are included in the article/Supplementary material, further inquiries can be directed to the corresponding author.
