# Supplementary figures and images for: Uncovering the connection between tunicamycin-induced respiratory deficiency and reduced fluconazole tolerance in Candida glabrata
Source: Front Microbiol. 2025 Apr 28;16:1528341. doi: 10.3389/fmicb.2025.1528341 (PMC12066676; doi:10.3389/fmicb.2025.1528341)

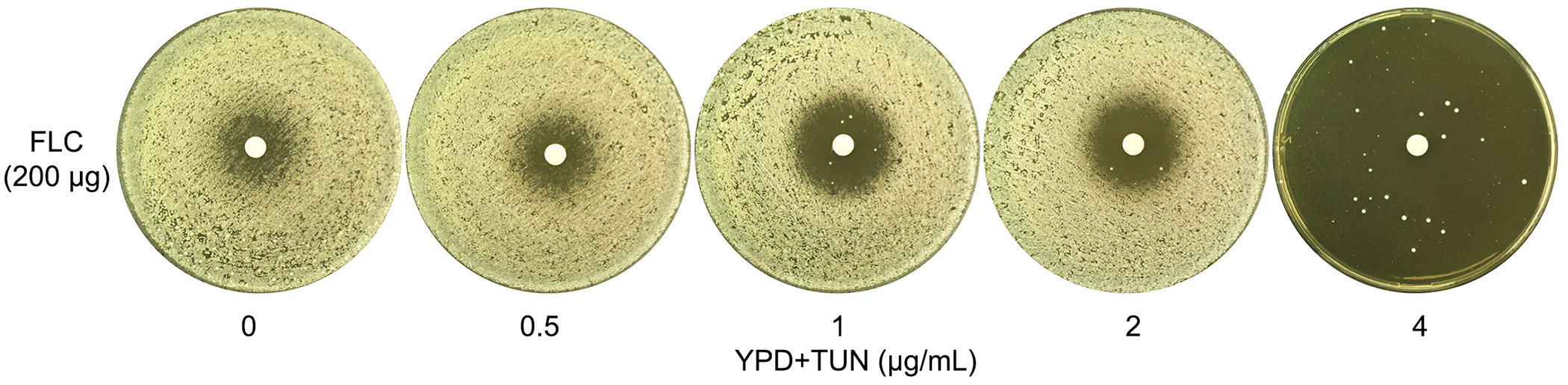

Supplement: Supplementary file 1 [file Image_1.tif]

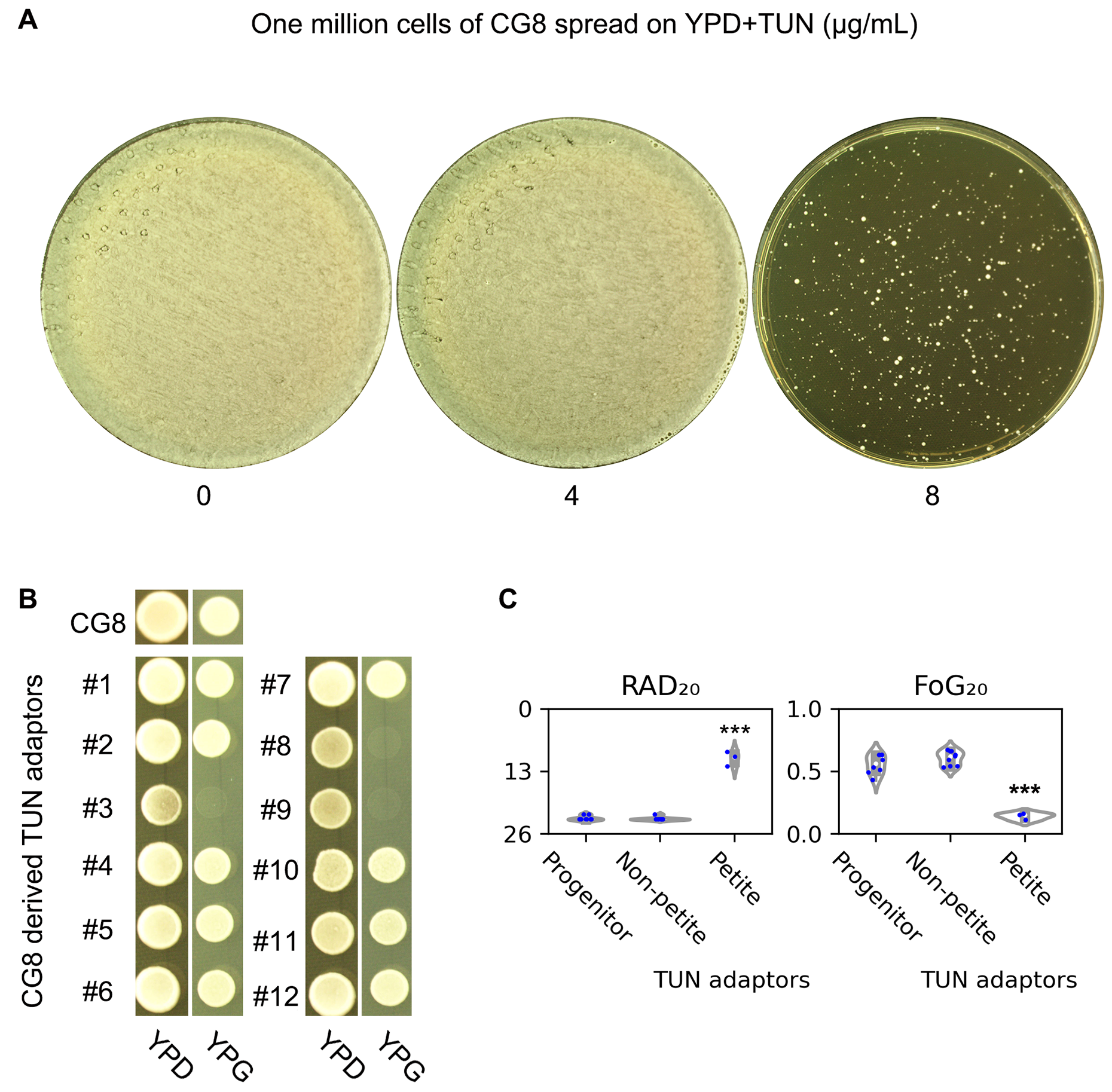

Supplement: Supplementary file 2 [file Image_2.tif]

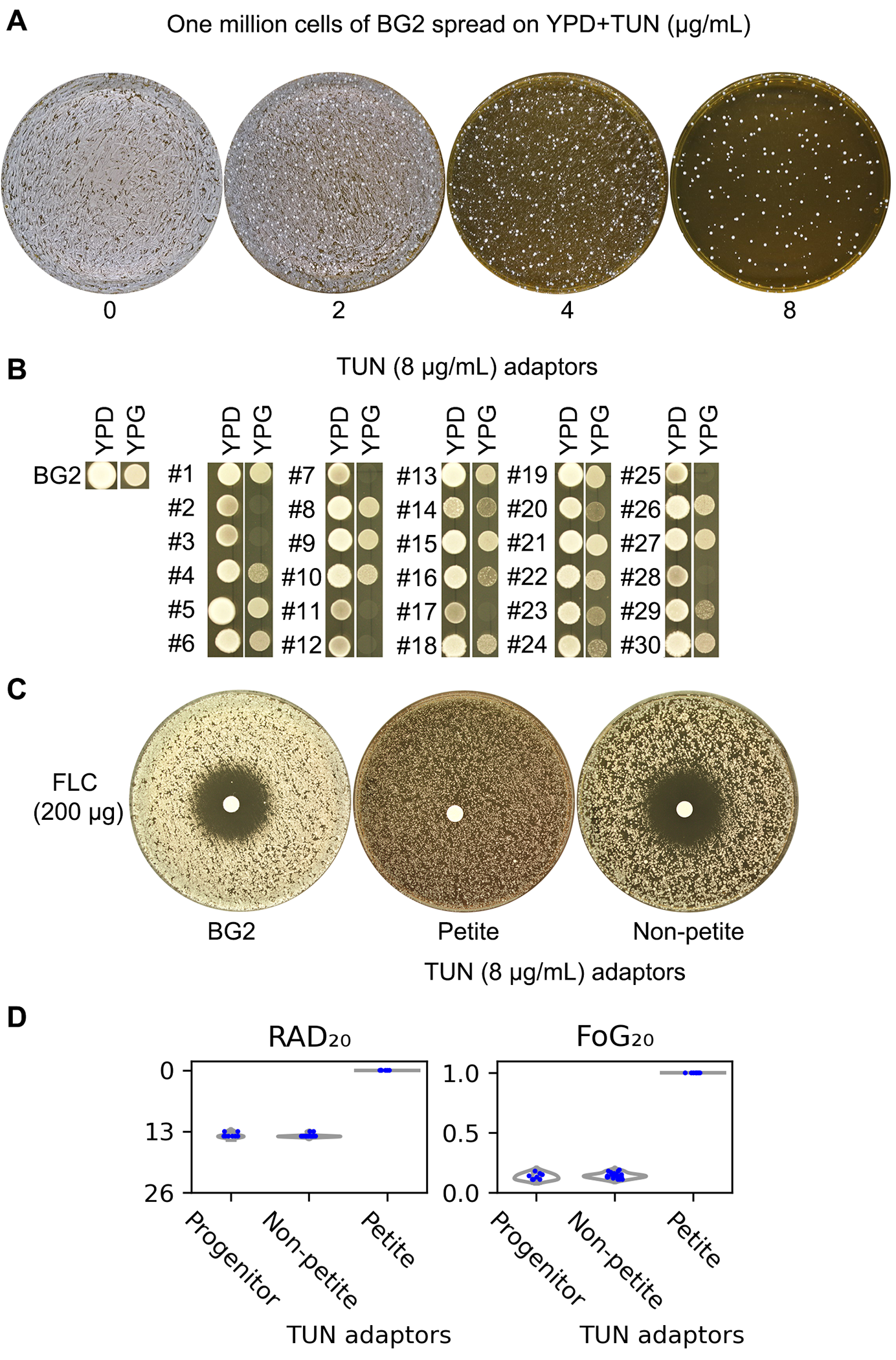

Supplement: Supplementary file 3 [file Image_3.tif]
