# Supplementary material for: Uncovering the connection between tunicamycin-induced respiratory deficiency and reduced fluconazole tolerance in Candida glabrata
Source: Front Microbiol. 2025 Apr 28;16:1528341. doi: 10.3389/fmicb.2025.1528341 (PMC12066676; doi:10.3389/fmicb.2025.1528341)
Supplement: Supplementary file 4 [file Table_1.docx]

Table S1. Primers used in this study

| Gene | Sequence |
| --- | --- |
| *ERG28* | Fw: TGTACATCACTGAACCCCACA |
|  | Rv: TCAGAGAAGTCGAAGCAACG |
| *ERG9* | Fw: CGGTATGGCCGACTACATCT |
|  | Rv: TCCTCGGCGTAGTCTCTGAT |
| *ERG1* | Fw: AACAACATCGACGCTTACCC |
|  | Rv: GGAATCTACCGTGGACCAGA |
| *ERG7* | Fw: CAAACAGATGCCTGATGGTG |
|  | Rv: GTTGCTGCCTGGACTTTAGC |
| *ERG11* | Fw: ACGGTACCAAGCCATACGAG |
|  | Rv: GAACACTGGGGTGGTCAAGT |
| *ERG24* | Fw: GGTAGCTATGCGTTGGCAAT |
|  | Rv: GTTGCCGGAGTTACCACCTA |
| *ERG25* | Fw: CTTGGCCAACATCGGTAAGT |
|  | Rv: GGTTGGTTGCAACTTCCACT |
| *ERG26* | Fw: CTTCACCTGTCCATGGAGGT |
|  | Rv: TAGGCATCCATTGGGACTTC |
| *ERG27* | Fw: GGATCCATGCTCTGTGGATT |
|  | Rv: GGGGTTGGTAACAGCTTCAA |
| *ERG6* | Fw: AAGGATCTTGCTGACGAGGA |
|  | Rv: ACCTTCGACCAAACCAACAG |
| *ERG2* | Fw: CAGCAATTGGGACTGAAGGT |
|  | Rv: CCGGGCATAGCATATTGTTT |
| *ERG3* | Fw: CACCCAGTCGACGGTTACTT |
|  | Rv: TTGACAACTGGGTTGTTGGA |
| *ERG5* | Fw: AGCGAGGACCAGATCAGAAA  Rv: CTTAGCTCTTTGGGCACAGG |
| *ERG4* | Fw: TATGGGAGCTCCATTGAACC |
|  | Rv: GGGCTAACATGACGACACCT |
| *PDR1* | Fw: AAAGGGAGTGACAGCGAGAA |
|  | Rv: ATGGCGTCAATGGATGATTT |
| *CDR1* | Fw: TGCAGGACCAAGTCAGACAG |
|  | Rv: CTCATCGGAAGTAGGGTCCA |
| *CDR2* | Fw: CGAGGAGGAAGACGACTACG |
|  | Rv: GCAGGTTCAGGAAAGTGCTC |
| *SNQ2* | Fw: ACGACCAATCAATGCAACAA |
|  | Rv: ACACCACCTCTGGAAAATGC |
| *ACT1* | Fw: TTGCCACACGCTATTTTGAG |
|  | Rv: ACCATCTGGCAATTCGTAGG |
|  |  |
|  |  |
