# Supplementary material for: Uncovering the connection between tunicamycin-induced respiratory deficiency and reduced fluconazole tolerance in Candida glabrata
Source: Front Microbiol. 2025 Apr 28;16:1528341. doi: 10.3389/fmicb.2025.1528341 (PMC12066676; doi:10.3389/fmicb.2025.1528341)
Supplement: Supplementary file 5 [file Data_Sheet_1.pdf]

Supplementary text

Supplementary Table S1. Sequences of primers used in this study

Supplementary Figure S1. Evaluation of the effect of different concentrations of tunicamycin on fluconazole tolerance

C. glabrata CG4 cells were spread on YPD plates supplemented with the indicated concentrations of tunicamycin (TUN). Disks containing 200 μg of fluconazole (FLC) were placed on the plates. The plates were incubated at 30°C for 48 hours and then photographed.

Supplementary Figure S2. Effect of tunicamycin on C. glabrata isolate CG8 and petite formation

(A) Approximately one million CG8 cells were plated on YPD plates supplemented with the indicated concentrations of TUN. (B) Twelve adaptors were randomly selected from the plate containing 8 μg/mL TUN and tested for growth on both YPD and YPG plates. For each adaptor, 3 μL of cells at a density of 1×10^6 cells/mL were spotted onto the plates. The plates were incubated at 30°C for 48 hours and then photographed. (C) The 12 adaptors (3 petites and 9 non-petites) were tested by disk diffusion assay with disks containing 200 μg FLC. For the CG8 progenitor, 8 individual colonies were tested as 8 biological replicates, and for each adaptor, 3 individual colonies were tested as 3 biological replicates. The data shown represent the means of the biological replicates.

Supplementary Figure S3. Effect of tunicamycin on C. glabrata reference strain BG2

(A) Approximately one million BG2 cells were spread on YPD plates supplemented with TUN and incubated at 30°C for 3 days before imaging. (B)

Thirty adaptors (#1–#30) were randomly selected from the plate containing 8 μg/mL TUN. The progenitor strain and adaptors were tested for growth on YPG plates, which were incubated at 30°C for 48 hours before imaging. (C) Disk diffusion assays were performed to compare FLC resistance between the progenitor strain and TUN adaptors. Disks contained 200 μg FLC. The progenitor and non-petite adaptors exhibited clear ZOI of similar size, while petite adaptors showed no detectable ZOI. (D) Disk diffusion assay images were analyzed using *diskImageR*. RAD_{20} and FoG_{20} values are shown for the progenitor strain (8 individual colonies), petite adaptors, and non-petite adaptors.
